# Assessing the Impact of Exercise on Quality of Life in Advanced-Stage Cancer Patients: A Systematic Review and Network Meta-Analysis of Randomized Controlled Trials

**DOI:** 10.3390/cancers17142329

**Published:** 2025-07-14

**Authors:** Yang-Yi Chang, Hung-Chun Hsiao, Ting-Wei Wang

**Affiliations:** 1 School of Medicine, National Yang-Ming Chiao Tung University, Taipei 30010, Taiwan; chang.yang.yi.g02@gmail.com (Y.-Y.C.);; 2 Chang Gung Memorial Hospital, Linkou 333423, Taiwan; 3 Institute of Biophotonics, National Yang-Ming Chiao Tung University, Taipei 30010, Taiwan; 4 Department of Computer Science, Whiting School of Engineering, Johns Hopkins University, Baltimore, MD 21218, USA

**Keywords:** advanced-stage cancer, quality of life (QoL), aerobic exercise, network meta-analysis, randomized controlled trials (RCTs), exercise interventions

## Abstract

**Background/Objectives** This systematic review and network meta-analysis aimed to determine the most effective therapeutic exercise modality for improving quality of life (QoL) in patients with advanced-stage cancer. Specifically, the study compared the effects of aerobic training, strength training, and combined aerobic and strength training on QoL outcomes. **Methods** A systematic literature search was conducted in PubMed, Embase, Cochrane Reviews, and the Cochrane Central Register of Controlled Trials up to 24 February 2023. The review adhered to PRISMA guidelines. Included studies were randomized controlled trials (RCTs) involving adult patients with advanced-stage cancers (e.g., pancreatic, colorectal, lung, breast, prostate, gastrointestinal, gynecological, hematological, head and neck, melanoma, or cancers with bone metastases). The primary outcome was post-intervention QoL, while the secondary outcome assessed was the dropout rate across exercise modalities. **Results** Aerobic training demonstrated the greatest improvement in QoL with a standardized mean difference (SMD) of 0.30 (95% CI: 0.00 to 0.61), followed by strength training (SMD = 0.13; 95% CI: −0.41 to 0.66) and combined training (SMD = 0.07; 95% CI: −0.11 to 0.24). However, none of the interventions showed statistically significant superiority. Dropout rates were comparable across all exercise modalities and control groups, suggesting strong adherence and feasibility of these interventions in advanced cancer populations. **Conclusions** While all exercise modalities were associated with improved QoL in patients with advanced-stage cancer, no single intervention emerged as significantly superior. Aerobic exercise may offer a slight advantage, although this effect was not statistically significant. These results highlight the importance of individualized exercise prescriptions based on patient preference, functional status, and treatment context. Further research is warranted to identify patient subgroups that may benefit most from specific exercise interventions and to explore QoL subdomains such as fatigue, emotional well-being, and physical functioning.

## Simple Summary

This study investigates which type of exercise—aerobic, strength training, or a combination of both—is most effective in improving quality of life (QoL) among patients with advanced-stage cancer. By analyzing data from 11 randomized controlled trials, we found that aerobic exercise may offer a slight advantage in improving QoL, though differences between exercise types were not statistically significant. Importantly, dropout rates were similar across all exercise groups, suggesting that each modality is generally well tolerated. These findings may help clinicians and patients tailor exercise plans according to individual preferences and needs.

## 1. Introduction

The outcomes of cancer survivors have progressively increased during the past decades, to the point where the 5-year survival of all cancers is now 69% [1]. This success is largely due to developments in medical screenings and therapies [2,3]. Nevertheless, with increasing survival rates after the cancer diagnosis, the QoL of survivors still represents a challenge. The challenges of survivors include monitoring comorbidities, side effects from treatment, and the cost of continued care. With respect to health problems, fatigue is a prominent problem among survivors’ health status [4]. Exercise addresses these issues through several biological mechanisms: it enhances cardiorespiratory fitness and skeletal-muscle oxidative capacity, reduces systemic inflammation, boosts immune surveillance, and triggers neuroendocrine changes (endorphin, serotonin, and dopamine release) that lessen anxiety, depression, and insomnia. Together, these effects translate into lower fatigue, better functional capacity, and higher perceived well-being.

This is particularly problematic for later-stage cancer patients who frequently undergo aggressive treatment with little corresponding support for coping with treatment side effects. In this way, these patients can have a poor quality of life related to their symptoms, such as weight loss, hair loss (alopecia), and treatment toxicity [5]. These results emphasize the need for integrative approaches to care that target not just the disease, but also its impact on a patient’s life more generally. Survivorship care plans are vital, particularly when they balance physical, emotional, and financial care for each patient.

Exercise-facilitated programs are increasingly considered an integral part of conventional cancer care, imparting physical functioning and psychological well-being as well as health-related QoL for survivors [6]. It is important to distinguish among related concepts: physical activity refers to any bodily movement produced by skeletal muscles that results in energy expenditure; therapeutic exercise denotes structured, repetitive, and goal-directed movements designed to restore or enhance physical function; and physical therapy involves clinical interventions administered by licensed professionals to address specific impairments, disabilities, or functional limitations. While exercise-based interventions have shown benefits in early-stage cancer populations, particularly in breast and prostate cancers [7], their efficacy in advanced-stage cancer remains less well established. This suggests a lack of evidence relating to the role of exercise in cancer at all stages, highlighting the importance of large research projects including a range of cancer types and stages.

Recent work is working on assessing the influence of exercise on the QoL of patients with advanced cancer with different natures. These endpoints have included chronic fatigue, functional status, dyspnea, and survival. Some reviews report a positive effect of exercise on QoL, fatigue, and dyspnea [5,7]. Nevertheless, there are still controversies due to the heterogeneity of the effects that could be explained by the different types of cancer models, exercise interventions, and clinical trial settings [8,9]. While the implication of the different exercise methods and frequencies for improved QoL has been described in many studies and reviews [10,11], there is also a need for evidence incorporating patients at all stages of cancer and specific to the nature of each type of cancer. To standardize terminology, we categorize interventions into four modalities: (i) aerobic training (continuous or interval activities at 40–85% VO_2_-peak), (ii) resistance training (muscle-strengthening at 50–80% 1-RM), (iii) combined aerobic + resistance programs, and (iv) mind-body/low-impact exercise such as yoga, tai chi, or qigong. Delivery mode (supervised vs. home-based) and exercise dose (frequency × intensity × time × type) will also be extracted for subgroup analysis.

This research uses network meta-analysis (NMA)—a method that enables the simultaneous comparison of multiple interventions—to address the current knowledge gap regarding the efficacy of exercise interventions on quality of life (QoL) in advanced cancer patients [12]. NMA has the advantage over standard systematic reviews and pairwise meta-analyses in that it allows the comparison of several exercise interventions simultaneously. This approach builds a full network of evidence, including direct comparisons among interventions and indirect comparisons across the studies. This systematic review and network meta-analysis aimed to identify the most effective exercise modalities for improving quality of life (QoL) in patients with advanced-stage cancer. Critically, no prior synthesis has ranked exercise interventions across different cancer types within this population. By mapping the full intervention network, this study provides a comparative analysis of aerobic, strength, and combined exercise strategies to inform evidence-based recommendations for supportive cancer care.

## 2. Materials and Methods

This study was performed in accordance with the Preferred Reporting Items for Systematic Reviews and Meta-Analysis (PRISMA) statement for network meta-analysis (PRISMA NMA) [13]. The registration number of this study is INPLASY202430068 [14]. Ethical approval and informed consent. This study is not applicable for approval of ethical review board or informed consent.

### 2.1. Database Searches and Study Identification

Two reviewers (YYC and TWW) independently searched PubMed, Embase, Web of Science, Cochrane Reviews, and Cochrane Central Register of Controlled Trials. They utilized same pre-defined search strategy: (Advanced cancer OR Metastasis OR Terminal illness) AND (End of life OR Palliative care) AND (Quality of life OR QoL) AND (Physical Activity OR Exercises OR Physical exercise OR Yoga OR Aerobic) AND (Random OR Randomized). This search sought studies for a systematic review and network meta-analysis, from the inception date for each database to 24 February 2024.

The titles and abstracts of the retrieved studies were screened independently at first by the two researchers for relevance according to a pre-specified consensus rule. This method was used on the mentioned databases to identify trials meeting the inclusion criteria. Moreover, the bibliographies of included review articles [5,7,8,9] were checked, and manual searches were performed to avoid any studies that may have been omitted. No language restrictions were used in the search strategy.

### 2.2. Inclusion and Exclusion Criteria

The systematic review was performed using the PICO model, where the following were determined: (1) Population: Cancer patients with advanced cancer (stage III–IV); (2) Intervention: Structured, exercise-based interventions, including aerobic training (e.g., walking, cycling), strength/resistance training (e.g., weightlifting, resistance bands), or a combination of both; (3) Comparison: A comparison group without interventions; and; (4) Outcome: Post-intervention quality of life, measured using validated instruments such as the EORTC QLQ-C30, FACT-G, or SF-36. The inclusion criteria for this analysis were as follows: (1) RCTs with patients diagnosed with advanced stage (III–IV) cancer, as classified in previous research [5]; (2) RCTs that examined the effects of exercise-based interventions on quantitative measures of quality of life; (3) Control groups with either no intervention or standard care; (4) Studies reporting quality of life outcomes prior to and post the intervention.

On the contrary, the exclusion criteria for the review and network meta-analysis were defined as follows: (1) studies for which no measure of quality of life was provided; (2) studies that focused specifically on functional domains of quality of life; (3) studies that did not provide baseline data; (4) studies lacking physical exercise-based interventions; (5) studies that excluded patients with advanced cancer diagnosis; (6) studies that were not based on a randomized controlled trial design; (7) studies with missing data; (8) studies that included patients previously treated with exercise interventions; (9) studies based exclusively on protocoled documentation.

### 2.3. Modelling for Network Meta-Analysis

To ensure accuracy, we strictly adhered to methodological standards during model formulation in this network meta-analysis. We limited comparisons to exercise versus exercise or exercise versus standard care to reduce the risk of heterogeneity. This decision excluded comparisons with nutritional supplements or pharmacological interventions to prevent divergent network structures and ensure consistent results. Two authors (YYC and TWW) categorized exercise types based on in-depth discussions about exercise prescriptions.

The network plot (netplot) visually depicts the structure of included comparisons across exercise modalities. Additionally, we present a netleague table to summarize the comparative effects and rankings of each intervention. In this table, treatments are ranked from best to worst along the leading diagonal. Estimates above the diagonal are derived from pairwise meta-analyses, while those below the diagonal are derived from the network meta-analysis, allowing for simultaneous evaluation of direct and indirect evidence across all interventions.

### 2.4. Methodological Quality Appraisal

To evaluate the methodological rigor of the studies incorporated into our analysis, we employed the Cochrane risk of bias tool for randomized trials (version 2, RoB 2, London, UK) [15]. This instrument critically examines six key dimensions to ascertain the quality of a study: the process of randomization, fidelity to the intervention, completeness of outcome data, accuracy of outcome measurement, the presence of selective outcome reporting, and the overarching risk of bias.

### 2.5. Primary Outcome: Quality-of-Life Improvement, Standardized Mean Difference

In this study, the main outcomes examined were changes in quality of life, measured using structured scales. The process for data extraction was carefully defined, with a focus on cancer-specific quality-of-life measurement tools, such as the European Organization for Research and Treatment of Cancer Quality-of-Life Questionnaire [16], followed by general quality-of-life assessment methods, including the Functional Assessment of Cancer Therapy-General [17] and the Short Form-36 Health Survey [18].

### 2.6. Secondary Outcome: Risk Difference of Dropout Rates

The secondary outcome metric was the difference in attrition rates, which was a clear measure. For example, if a participant chooses a specific exercise program designed to improve their quality of life and experiences a 15% attrition rate, while the control group, which receives standard care, shows an 11% attrition rate, this may lead some individuals in the control group to start an exercise program on their own. Therefore, the difference in attrition rates between these groups is calculated to be 4%.

### 2.7. Statistical Analyses

The diversity of exercise interventions necessitated using a random-effects model for the network meta-analysis [19]. This analysis was conducted using MetaInsight (version 4.0.2, Complex Reviews Support Unit, National Institute for Health Research, London, UK), which utilizes the netmeta package in R for frequentist statistical analysis [20].

Forest plots and a network plot were created to display the pairwise comparisons from individual studies and facilitate the interpretation of the data. Subsequently, forest plots were used to present the standardized mean differences in quality-of-life improvements and the variance in dropout risks among different exercise types compared to a control group. These outcomes were summarized with point estimates and 95% confidence intervals (95% CI) [21]. The assessment of exercise types included direct and indirect comparison results, presented with numerical estimates. Inconsistency analyses were performed to check for data discrepancies. A two-tailed *p*-value of less than 0.05 was considered statistically significant.

### 2.8. Sensitivity Analyses

We conducted two sensitivity analyses to improve the reliability of our study’s findings. The first analysis used a leave-one-out approach to ensure no single study unduly influenced the overall results. By systematically removing one study at a time, we assessed the stability of our conclusions regarding quality-of-life improvements.

The second analysis focused on the effect of different pre-post correlation coefficients on the calculated mean and standard deviation of changes in quality-of-life scores. Following the Cochrane Handbook’s recommendation, we initially assumed a pre-post correlation coefficient of 0.8 [22]. Recognizing the debate over the optimal coefficient, with values like 0.5 and 0.7 also common [23], we performed a sensitivity analysis using a coefficient of 0.5 to examine its impact on our results [23]. This included an evaluation of the effect’s direction, magnitude, statistical significance, and ranking.

### 2.9. Publication Bias

Additionally, we thoroughly assessed the risk of publication bias following the Cochrane Handbook for Systematic Reviews of Interventions [12]. This involved creating a funnel plot with StataCorp LLC, College Station, TX (USA), Version 18.0. and conducting an Egger’s regression test to quantitatively determine the presence of significant publication bias, especially regarding comparisons with the control group.

## 3. Results

### 3.1. Study Identification and Network Model Formation

**Figure 1:**
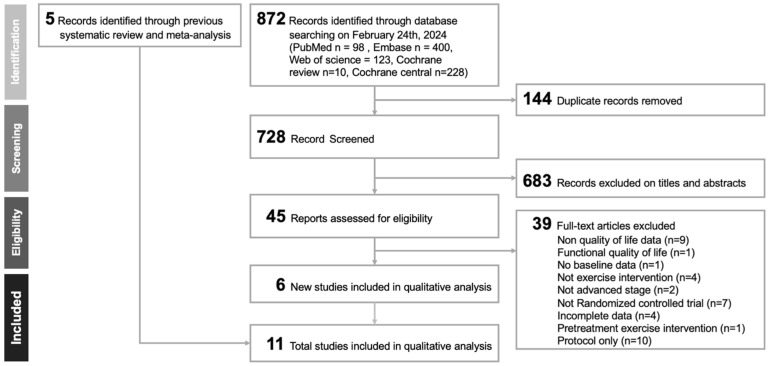
Flow diagram depicting the study selection process in accordance with the PRISMA (Preferred Reporting Items for Systematic Reviews and Meta-Analyses) guidelines.

Figure 1 illustrates the study selection process in accordance with the PRISMA guidelines. The PRISMA-NMA extension checklist is provided in Supplementary Table S1 and Supplementary Table S2 details the number of records retrieved from each database. Following the removal of duplicates and initial screening of titles and abstracts for relevance, eleven randomized controlled trials [24,25,26,27,28,29,30,31,32,33,34] met the inclusion criteria and were included in the final analysis. A comprehensive list of excluded full-text articles, along with the specific reasons for exclusion, is available in Supplementary Table S3.

**Figure 2:**
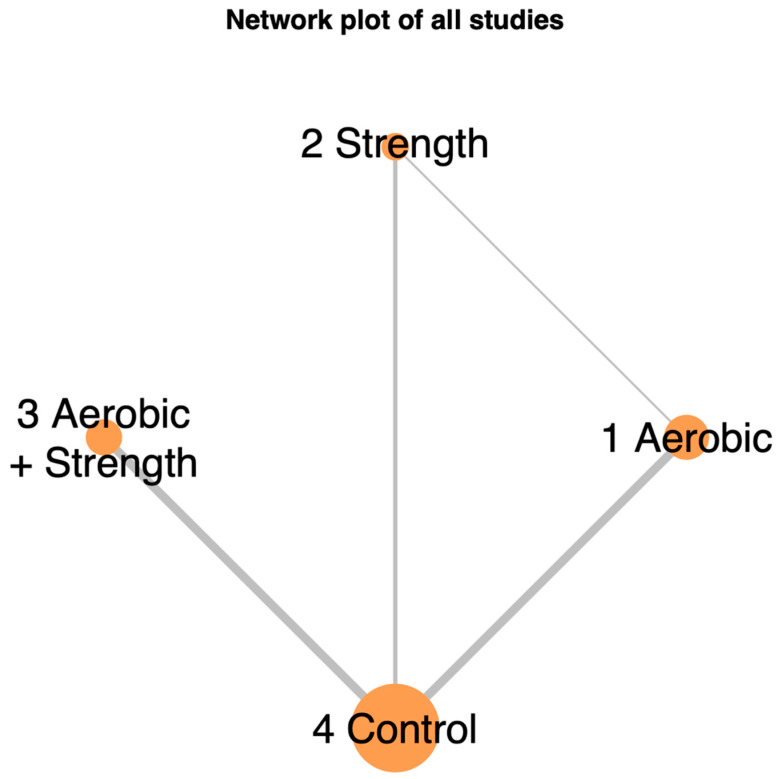
Network illustrating the comparative effects of different exercise interventions on quality of life in advanced-stage cancer patients. The size of each node and the thickness of each connecting line reflect the number of trials contributing to each intervention and comparison.

Our study analyzed eleven randomized controlled trials covering 699 participants. We categorized the physical activities from these studies into three groups: concurrent aerobic and strength training, aerobic-only activity, and strength-only exercise. Figure 2 in the network model graphically depicts these exercise modalities and their respective interventional approaches.

Figure 2 in the network model graphically depicts these exercise modalities and their respective interventional approaches. The studies covered a range of cancer types: three on pancreatic cancer, one on colorectal cancer, two on lung cancer, one on breast cancer, one on prostate cancer, and three on various cancers, cited as follows: pancreatic [34], colorectal [24], lung [29,31], breast [30], prostate [33], and mixed [25,26,27,28,31]. Table 1 provides detailed inclusion criteria, study locations, participant mean age with standard deviation, exercise intervention types, quality-of-life assessment tools, and attrition rates.

### 3.2. Methodological Quality of the Included Studies

In our analysis, only 9.1% (1 out of 11) of the studies demonstrated a low risk of bias. In contrast, 63.6% (7 out of 11) showed a moderate risk of bias, and 27.2% (3 out of 11) had a high risk of bias, as shown in Figure S1. The most frequent issues were deviations from intended interventions (Domain 2), incomplete outcome data (Domain 3), and selective reporting (Domain 5). The studies with a moderate risk of bias had inconsistencies in their protocols across different study arms, which could affect participant adherence and the effectiveness of the interventions. Detailed evaluations of the risk of bias are provided in Table S4.

### 3.3. Primary Outcome: Aerobic Training Most Effective

**Figure 3:**
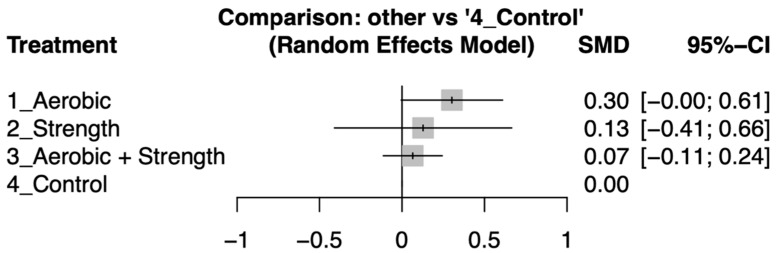
Forest plot illustrates the standard mean difference (SMD) in quality-of-life improvements between exercise intervention and control groups. The plot highlights the variability in effect sizes across the groups, showcasing the comparative effectiveness of various exercise interventions on participants’ quality of life.

Aerobic training showed a modest improvement in quality-of-life metrics, with an effect size of 0.3 (95% Confidence Interval [CI]: −0.00 to 0.61), suggesting a potential but uncertain benefit. Strength training alone yielded a small effect size of 0.13 (95% CI: −0.41 to 0.66), indicating a slight, albeit statistically insignificant, improvement. The combination of aerobic and strength training resulted in an effect size of 0.07 (95% CI: −0.11 to 0.24), and strength training alone had an effect size of 0.19 (95% CI: −1.08 to 1.46). Neither combination nor isolated strength training significantly differed from the control group, as depicted in Figure 3. For a detailed analysis of the comparisons between study groups, refer to Figure S2.

Exercise interventions were ranked based on their effect sizes for improving quality of life, with aerobic training showing the greatest efficacy, followed by strength training, and then combined aerobic and strength training. For a detailed comparison and ranking, see netleague table (Table 2).

### 3.4. Secondary Outcome: Comparable Dropout Rates

**Figure 4:**
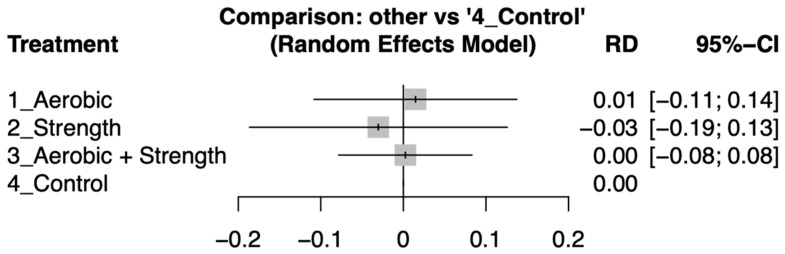
Forest plot showing the risk differences (RDs) in dropout rates between various exercise interventions and control groups among patients with advanced-stage cancer. Confidence intervals overlapping zero indicate no statistically significant differences in attrition.

Dropout rates across the various exercise modalities were similar to those in the control groups, as indicated by risk differences with 95% confidence intervals that included zero (Figure 4). Detailed pairwise comparisons of dropout rates between intervention arms are provided in Supplementary Figure S3.

### 3.5. Inconsistency Test

To assess consistency within the network, we constructed the network geometry using intervention nodes and evaluated both direct and indirect comparisons. Inconsistency in quality-of-life outcomes is reported in Supplementary Table S5, and inconsistency related to dropout rates is shown in Supplementary Table S6. All comparisons yielded *p*-values greater than 0.05, indicating no statistically significant inconsistency between direct and indirect evidence.

### 3.6. Sensitivity Analyses

The one-study exclusion method’s sensitivity analysis showed consistent rankings and clinical importance across different physical activity categories. The analysis revealed that aerobic training significantly improved the quality of life for advanced-stage cancer patients. In contrast, resistance exercises and combined aerobic and resistance training had no significant impact. These findings are supported by Figure S4a–k.

Further sensitivity analysis confirmed the original results, where the correlation coefficient for pre- and post-intervention assessments was adjusted from 0.8 to 0.5, followed by a revised network meta-analysis (see Figure S5). This indicates that the conclusions regarding effect sizes, rankings, and interpretations remain valid even when initial assumptions are altered, as shown in Figure 3.

Overall, the results from these analyses verify the stability and credibility of our findings, highlighting that they are not affected by selective study exclusion or inclusion, nor by changes in analytical assumptions.

### 3.7. Publication Bias

Refer to Figure S6 for the funnel plot, which illustrates the analysis outcomes. The Egger’s test produced a *p*-value of 0.06, indicating the absence of significant publication bias.

## 4. Discussion

### 4.1. Summary of Main Results

Our network meta-analysis revealed that in advanced-stage cancer patients, all three exercise modalities (aerobic only, strength only, and strength) improved their quality of life to a limited extent compared to the control. Furthermore, all three groups showed statistically similar dropout rates compared to the control. Upon inter-arm comparison via standard mean difference (SMD), the aerobic-only exercise demonstrated the highest effectiveness (effect size: 0.30, 95% CI:0.00 to 0.61) followed by strength-only exercise (effect size: 0.13, 95% CI: −0.41 to 0.66) and lastly aerobic and strength exercise (effect size: 0.07, 95% CI: −0.11 to 0.24).

Our findings underscore the potential of consistent and supervised aerobic exercise as equally, if not more, effective than strength-based exercise alone or a combination of aerobic and strength training. This aligns with previous studies suggesting that exclusive aerobic exercise may mitigate certain extrinsic factors affecting exercise adherence, such as the location of rehabilitation centers and the required knowledge of exercise [33]. Additionally, home or neighborhood-based aerobic exercise [35] may offer a cost-effective alternative for multidisciplinary teams with limited resources, thereby reducing the barriers associated with strength training equipment, potential injuries, and spatial concerns [36].

### 4.2. Relationship to Current Literature

Exercise’s role in cancer prevention and treatment has been extensively studied, with numerous investigations linking exercise to improved immune response, vasculature modeling, and epigenetic modifications [37]. While past research has demonstrated improvements in quality of life across different cancer types [5,7,9,38], the significance of these findings has varied because of scarce high-quality trials, frequent lack of blinding, and wide heterogeneity in tumor biology and comorbidity profiles [5]. Furthermore, the optimal modality and frequency of exercise in advanced-stage cancer remain unclear [39]. The few RCTs that attempted direct comparison of different exercise modalities only looked at specific cancers, such as breast [40], prostate [41], and gastrointestinal [26] cancers, and, except for the last study, excluded advanced-stage patients. Thus, a gap persists in the current evidence, warranting a broader exploration of exercise’s impact on the quality of life in the advanced-stage population.

Traditional meta-analyses in the literature have faced challenges in comparing multiple interventions due to an inability to correct for variability [5,38]. This challenge stems from the diversity of cancer types and comorbidities, which might have confounded the effects of a single treatment during comparisons. However, recent advancements in network meta-analysis, as demonstrated by Wang et al. [11], offer a promising approach to address these limitations. Their study examined nine RCTs to compare exercise interventions in early-stage breast cancer survivors [11], yielding a significant inter-arm effect size and ranking using a random effects model. Regimented, supervised aerobic and strength exercise was shown to be most effective in improving the quality of life in early breast cancer [11]. Building on that framework, we applied NMA across 11 RCTs spanning seven tumour sites, thereby providing (to our knowledge) the first cross-cancer ranking of exercise modalities in an advanced-stage population.

Site-specific signals emerged. In the three pancreatic-cancer trials (all RoB, some concerns), aerobic elements produced small, concordant gains (SMD ≈ +0.20). Lung cancer studies (both RoB high) again favoured aerobic training (+0.15 to +0.18), but wide CIs reflected high attrition. Conversely, a cachectic mixed-tumor study with high risk of bias showed a neutral effect. These patterns suggest that symptom burden and protocol fidelity, indexed by our RoB grading, modulate observable benefit and help explain the overall modest pooled effects. Thirty-nine full-text articles were excluded after a detailed review (Supplementary Table S3). The main reasons were: no global QoL endpoint (*n* = 9), functional-only QoL scale (*n* = 1), no baseline data (*n* = 1), non-exercise comparator (*n* = 4), not randomised controlled trial (*n* = 7), not advanced stage (*n* = 2), incomplete data (*n* = 4), pretreatment/prehabilitation intervention (*n* = 1) and protocol-only/feasibility design (*n* = 10). None of these studies reported a clinically important QoL change, so their exclusion is unlikely to bias our estimate, but the sheer number underscores the paucity of rigorous late-stage trials.

Our aerobic-favoured ranking contrasts with Wang et al. in early-stage breast cancer [11], where combined aerobic + strength topped the league; this divergence highlights potential stage-specific physiology and indicates that advanced patients may prioritise low-skill, easily supervised modalities.

Participation adherence remains a challenge in exercise interventions for cancer patients, especially in advanced cancer. Our study highlighted significantly similar dropout rates compared to the control, with an extremely low to negative risk difference (RD), suggesting low dropout rates overall. However, this comparison was analyzed within the context of poor adherence in all studies’ intervention and control arms, with only Tsianakas et al. [26] reaching adherence of more than 90%. As a patient’s disease progresses, the accumulation of physiological aging, oncological side effects, chronic fatigue, declining physical function, and psychosocial burden proves formidable in reducing exercise motivation [35,42]. Studies on adherence behavior have shown that participation is contingent on pre-diagnosis level of physical activity [35] and professional supervision [40]; patients with higher levels of baseline physical activity and who received center-based or phone/video call guidance during intervention were more likely to complete their treatment [35].

### 4.3. Possible Explanations for Inter-Arm Differences

While we anticipated all three modalities to be effective, surprisingly, inter-arm analysis shows aerobic exercise as having a greater effect than aerobic and strength, with strength-only exercise, although with limited significance. The benefit of aerobic exercise is one of the most widely studied exercise modalities in cancer [37]. Sustained aerobic exercise is commonly linked to improved chronic fatigue, sleep quality, patient autonomy, and adherence [5,10,43]. It also triggers modifications of the inflammation response and vascular reperfusion. The increased recruitment of tumor inhibitory factors, such as nitric oxide (NO), enhances tumor cell apoptosis [44], while improved perfusion may allow for more efficient chemotherapy delivery [45]. The factors thus lead to improved symptom relief and treatment response, improving a patient’s quality of life. However, the underlying mechanism of aerobic exercise requires further exploration, since most studies focus on animal models with an underwhelming consensus on important mechanisms such as increased vascular perfusion [46].

Another possible factor for our inter-arm outcome might be patients’ preference for aerobic exercise. Studies on adherence have shown aerobic exercise, such as walking, to be more favorable among cancer patients [47] at a moderate intensity, with various exercises involved. In terms of location, home-based interventions were preferred by older adults [35,48], who also make up most of the cancer population. Despite established patient preferences and professional supervision, our analyzed study’s adherence to aerobic exercise remains statistically similar to other exercise modalities. This discrepancy underscores the complex, multifactorial nature of adherence in advanced-stage cancer. Despite being influenced by similar factors as the healthy population [36], advanced-stage cancer patients face additional psycho-social and disease burden that translates into poor motivation and worse quality of life.

While our study showed a potential for aerobic therapy in advanced-stage cancer, a discrepancy of significance was reflected in our study’s limited effect size and statistically non-significant 95% CI. The limited significance of aerobic exercise could be explained by underlying pathophysiological differences among cancer subtypes and staging. Different tumor biologies, the progressively evolving tumor microenvironment, and patient psychosocial well-being would impact how each patient responds to exercise. Furthermore, it cannot be discounted that due to the complexity behind the quality-of-life subdomains (emotional, physical, cognitive), the beneficial effect of exercise cannot be completely separated from effective cancer treatment [26]. However, our outcome could help narrow down which specific exercise modality to include in advanced cancer RCTs. Furthermore, we believe more studies could focus on how and to what extent exercise impacts specific QoL subdomains and explore the confounding effect of molecular subtypes for each cancer type and disease stage.

### 4.4. Strengths and Limitations

We believe this network meta-analysis had several strengths: (i) this is the first study, to the best of our knowledge, that compared different exercise modalities regardless of cancer type in advanced-stage cancer; (ii) the exclusive analysis of RCTs contributed to the robustness of comparative effectiveness assessments across different interventions; (iii) the use of a network analysis enables the incorporation of diverse cancer types, mitigating heterogeneity concerns—the use of a random effects model aids in drawing more generalizable conclusions by accounting for variations within and between studies.

Conversely, the study was also limited by (i) the small number of eligible RCTs, poor adherence overall, and a small sample size. The former limits the robustness and generalizability of conclusions drawn, while the latter impacts the overall effectiveness of exercise intervention; (ii) heterogeneity in the intervention time (i.e., treatment or post-treatment). We believe that standardizing the intervention period across studies could provide more uniform and comparable results; (iii) most participants were over 60 years old; (iv) certainty is constrained because nearly one-third of the included trials were at high risk of bias and only one met all low-risk criteria; (v) inconsistent reporting of comorbidities, ECOG status, cancer staging and cancer type across studies limited our ability to include detailed population data or conduct meaningful subgroup analyses. The overrepresentation of older participants may restrict the generalizability of findings to a broader age range, as younger individuals with advanced cancer could exhibit different responses to exercise interventions since psycho-social experiences and preferences may differ between age groups [49].

## 5. Conclusions

Our network meta-analysis comparing different exercise modalities in advanced-stage cancer patients revealed that aerobic exercise positively impacted quality of life more than combined aerobic and strength with strength exercises alone.

## Figures and Tables

**Table 1:** Summary of the included trials investigating exercise’s effect on improving quality of life in breast cancer survivors.

First Author	Study Country	Enrolled Population (Age)	Age	Participants in Nodes	QoL Scale (Range)	Week	QoL Improvement	Dropouts	Exercise Details
Neuzillet et al. (2023) [34]	France	Previously untreated, locally advanced, or metastatic pancreatic ductal adenocarcinoma, were fit for chemotherapy	62.8 ± 11.1	Aerobic + Strength 87 Control 85	EORTC QLQ-C30 (0~100)	16	8.2 ± 12.59 7.3 ± 13.37	87/156 85/157	The 16-week intervention combined personalized aerobic exercises (walking, Nordic walking, or cycling) and resistance training with elastic bands, tailored based on initial fitness assessments and adapted in duration, frequency, and intensity, with weekly remote supervision by an APA professional and participation of a volunteer physical activity partner.
Zimmer et al. (2018) [24]	Germany	Metastatic colorectal cancer	67.9 ± 8.8	Aerobic + Strength 15 Control 9	FACT-G (0~108)	12	(−0.63 ± 9.77 4.63 ± 7.68)	15/17 9/13	The intervention, supervised by a qualified sports therapist twice weekly at a sports center, included a 60 min program with three phases: Phase I focused on balance and coordination, Phase II on endurance and resistance training, and Phase III on cool down activities, adapting the difficulty to each participant’s capacity with continuous heart rate monitoring.
Grote et al. (2018) [25]	Germany	Participants were individuals diagnosed with cancer affecting the lung, pancreas, esophagus, head and neck, colon, rectum, or anus, and who were identified as being in a state of cachexia or pre-cachexia.	60.9 ± 13.1	Strength 10 Control 10	FACT-G (0~108)	15	(−15.7 ± 11.59 −16.2 ± 16.1)	10/10 10/10	The intervention, aimed at hypertrophic adaptation and supervised by a physiotherapist, involved a step-by-step submaximal approach with 3 sets of 8–12 repetitions across three core exercises—leg press, lat pull-down, and chest press—after a 5 min warm-up on an ergometer.
Tsianakas et al. (2016) [26]	UK	Participants had a confirmed diagnosis of recurrent or metastatic cancer, including breast, colorectal, upper gastrointestinal, gynecological, hematological, head and neck, melanoma, or prostate malignancies.	65.6 ± 10.8	Aerobic 13 Control 14	FACT-G (0~108)	12	(−2± 3.40 −2 ± 5.50)	13/21 14/21	The CanWalk intervention, a 12-week program designed to promote physical activity among participants, encouraged walking for at least 30 min every other day, supplemented with a motivational interview, printed materials, and optional participation in a national network of walking groups, with adherence and motivation supported by researchers trained in motivational interviewing techniques.
Jensen et al. (2014) [27]	Germany	Patients diagnosed with advanced gastrointestinal malignancies—including gastric, colorectal, pancreatic, and biliary tract cancers—were included in the study.	55 ± 13.1	Aerobic 11 Strength 10	EORTC QLQ-C30 (0~100)	12	(14.5± 28.18 13.3 ± 4.77)	11/13 10/13	Patients were randomized to undergo a 12-week program of either resistance exercise training (RET), focusing on large muscle groups with sessions including warm-up, strength exercises at 60–80% 1-RM, and cool-down, or aerobic exercise training (AET), progressively increasing from 60% to 80% of maximum pulse rate over 45 min sessions on a bicycle ergometer, both employing Borg’s Rating of Perceived Exertion Scale to adjust intensity.
Cheville et al. (2012) [28]	America	Pathology-confirmed Stage IV lung and colorectal cancers	64.7 ± 10.8	Aerobic + Strength 26 Control 30	FACT-G (0~108)	12	(1.07± 11.6 0.12 ± 10.22)	26/33 30/33	The intervention involved an initial 90-min one-on-one REST session with a pedometer-based walking program, subsequent bimonthly phone calls for progress review, resistance bands for graded exercises, and a calendar for tracking, with participants performing upper and lower body routines twice a week and gradually increasing repetitions, all monitored by two PTs and supported by pedometers to encourage brisk walking at least four days a week.
Hwang (2012) [29]	Taiwan	Patients with a confirmed diagnosis of adenocarcinoma for a duration exceeding four weeks were included.	60.0 ± 6.2	Aerobic 13 Control 11	EORTC QLQ-C30 (0~100)	12	(5.1± 7.79 3.1 ± 8.95)	11/13 7/11	The exercise intervention consisted of 24 sessions where participants in an outpatient clinic performed interval training three times a week on a treadmill or cycling ergometer, with high-intensity bursts and moderate active recovery, overseen by a physical therapist who adjusted the program biweekly based on individual responses, while monitoring vital signs and recording any adverse events.
Ligibel et al. (2016) [30]	America	Patients diagnosed with metastatic breast cancer or with locally advanced disease deemed unsuitable for surgical resection were eligible for inclusion.	50.0 ± 8.5	Aerobic 33 Control 43	EORTC QLQ-C30 (0~100)	16	(6± 17.5 −1 ± 21.5)	33/47 43/51	The 16-week exercise intervention consisted of moderate-intensity aerobic exercise, guided by both in-person and weekly telephone sessions with an exercise physiologist focusing on self-efficacy and safe practices, aiming for 150 min of exercise per week, with participants receiving a heart rate monitor, pedometer, exercise journal, and gym membership to track and facilitate their activity.
Henke et al. (2014) [31]	Germany	Patients with a diagnosis of non-small cell lung cancer (NSCLC) or small cell lung cancer (SCLC) at stage IIIA, IIIB, or IV, who had received inpatient palliative chemotherapy with a platinum-based regimen, were included in the study.	NR	Aerobic 18 Control 11	EORTC QLQ-C30 (0~100)	12	(5.73± 13.1 −6.41± 18.29)	18/25 11/21	During chemotherapy, patients engaged in a three-component training program: functional endurance exercises (hallway walking and stair climbing) 5 days a week, strength training every other day, and physiotherapeutic breathing techniques, all tailored to individual capacities and adjusted for safety, with physiotherapist supervision ensuring proper execution and intensity based on heart rate and dyspnea perception, and additional conventional physiotherapy as needed for dyspnea or joint issues.
Adamsen et al. (2009) [32]	Denmark	Participants were eligible if they had a confirmed cancer diagnosis and had completed at least one cycle of chemotherapy, either for advanced disease or as part of adjuvant therapy.	47.2 ± 10.6	Aerobic + Strength 118 Control 117	EORTC QLQ-C30 (0~100)	6	(3.4± 13.11 3.1± 14.17)	118/135 117/134	Participants in the intervention group underwent a rigorous six-week multimodal exercise regimen totaling 43 MET hours weekly at a hospital fitness facility, including high-intensity physical and relaxation training, body awareness, and massage, with progression monitored via bi-weekly one repetition maximum tests and continuous heart rate monitoring, while the control group received standard care with the option to join the exercise program post-study.
Cormie et al. (2013) [33]	Australia	With bone metastatic disease secondary to prostate cancer	72.2 ± 7.1	Strength 8 Control 7	SF-36 (0~100)	12	(−1.1± 6.26 −0.7 ± 5.44)	8/10 7/10	The 12-week exercise intervention consisted of twice-weekly, supervised 60 min resistance sessions targeting major muscle groups, with exercises adjusted for bone metastases to minimize force on affected areas. Participants aimed for 12–8 RM over 2–4 sets, progressively increasing load based on individual response, supplemented by at least 150 min of home-based moderate-intensity aerobic exercise weekly.

Abbreviation: EORTC QLQ-C30, European Organization for Research and Treatment of Cancer Quality of Life Questionnaire-Core 30; FACT-G, Functional Assessment of Cancer Therapy-General; SF-36, Short Form-36 Health Su.

**Table 2:** Netleague table showing pairwise comparisons and ranking of exercise interventions for improving quality of life in patients with advanced-stage cancer.

**Aerobic**	−0.05 [−0.91, 0.80]	.	0.33 [0.01, 0.65]
0.17 [−0.38, 0.73]	**Strength**	.	−0.01 [−0.67, 0.65]
0.24 [−0.12, 0.59]	0.06 [−0.50, 0.63]	**Aerobic + Strength**	0.07 [−0.11, 0.24]
0.30 [0.00, 0.61]	0.13 [−0.41, 0.66]	0.07 [−0.11, 0.24]	**Control**

Estimates derived from pairwise meta-analyses are presented above the diagonal, while those from network meta-analyses appear below the diagonal.

## Data Availability

Data are contained within the article and Supplementary Files.

## Supplementary Materials

The following supporting information can be downloaded at: https://www.mdpi.com/article/10.3390/cancers17142329/s1, Table S1: PRISMA NMA checklist; Table S2: Keywords and search results in different databases; Table S3: Excluded articles and reasons; Table S4: Detailed quality assessment of included studies; Table S5: Inconsistency test results of standardized mean difference in quality of life improvement for advanced-stage cancer patients after exercise; Table S6: Inconsistency test results for risk difference of dropout rates after exercise intervention; Figure S1: Summary of quality assessment for the studies included using version 2 of the Cochrane risk-of-bias tool; Figure S2: Forest plot of pair-wise comparisons showing standardized mean differences (SMDs) for quality of life improvement in advanced-stage cancer survivors; Figure S3: Forest plot of pair-wise comparisons showing risk differences (RDs) of dropout rates in advanced-stage cancer survivors—none of the comparisons reached statistical significance; Figure S4: Sensitivity analysis using the one-study removal method, showing robustness of clinical implications; Figure S5: Sensitivity analysis using a pre-post correlation coefficient of 0.5 (vs. 0.8), showing unchanged ranking and interpretation of SMDs; Figure S6: Funnel plot of all paired comparisons involving the common comparator (control group); Egger’s test yielded *p* = 0.06, indicating no significant publication bias.
