# Supplementary material for: Assessing the Impact of Exercise on Quality of Life in Advanced-Stage Cancer Patients: A Systematic Review and Network Meta-Analysis of Randomized Controlled Trials
Source: Cancers (Basel). 2025 Jul 14;17(14):2329. doi: 10.3390/cancers17142329 (PMC12293842; doi:10.3390/cancers17142329)
Supplement: Supplementary file 1 [file cancers-17-02329-s001.zip › cancers-3727377-supplementary.pdf]

Table S1. PRISMA NMA Checklist.

Section/Topic	Item #	Checklist Item	Reported on Page #
**TITLE**			
Title	1	Identify the report as a systematic review *incorporating a network meta-analysis (or related form of meta-analysis).*	Title
**ABSTRACT**			
Structured summary	2	Provide a structured summary including, as applicable: **Background:** main objectives **Methods:** data sources; study eligibility criteria, participants, and interventions; study appraisal; and *synthesis methods, such as network meta-analysis.* **Results:** number of studies and participants identified; summary estimates with corresponding confidence/credible intervals; *treatment rankings may also be discussed. Authors may choose to summarize pairwise comparisons against a chosen treatment included in their analyses for brevity.* **Discussion/Conclusions:** limitations; conclusions and implications of findings. **Other:** primary source of funding; systematic review registration number with registry name.	Abstract
**INTRODUCTION**			
Rationale	3	Describe the rationale for the review in the context of what is already known, *including mention of why a network meta-analysis has been conducted.*_	Introduction
Objectives	4	Provide an explicit statement of questions being addressed, with reference to participants, interventions, comparisons, outcomes, and study design (PICOS).	Introduction
**METHODS**			
Protocol and registration	5	Indicate whether a review protocol exists and if and where it can be accessed (e.g., Web address); and, if available, provide registration information, including registration number.	Materials and Method
Eligibility criteria	6	Specify study characteristics (e.g., PICOS, length of follow-up) and report characteristics (e.g., years considered, language, publication status) used as criteria for eligibility, giving rationale. *Clearly describe eligible treatments included in the treatment network, and note whether any have been clustered or merged into the same node (with justification).*_	Materials and Method / Inclusion and exclusion criteria
Information sources	7	Describe all information sources (e.g., databases with dates of coverage, contact with study authors to identify additional studies) in the search and date last searched.	Table S2
Search	8	Present full electronic search strategy for at least one database, including any limits used, such that it could be repeated.	Table S2
Study selection	9	State the process for selecting studies (i.e., screening, eligibility, included in systematic review, and, if applicable, included in the meta-analysis).	Materials and Method/ Data extraction

Data collection process	10	Describe method of data extraction from reports (e.g., piloted forms, independently, in duplicate) and any processes for obtaining and confirming data from investigators.	Materials and Method / Data extraction
Data items	11	List and define all variables for which data were sought (e.g., PICOS, funding sources) and any assumptions and simplifications made.	Materials and Method
**Geometry of the network**	**S1**	Describe methods used to explore the geometry of the treatment network under study and potential biases related to it. This should include how the evidence base has been graphically summarized for presentation, and what characteristics were compiled and used to describe the evidence base to readers.	Materials and Method / Modeling for network meta- analysis
Risk of bias within individual studies	12	Describe methods used for assessing risk of bias of individual studies (including specification of whether this was done at the study or outcome level), and how this information is to be used in any data synthesis.	Materials and Method / Quality appraisal
Summary measures	13	State the principal summary measures (e.g., risk ratio, difference in means). *Also describe the use of additional summary measures assessed, such as treatment rankings and surface under the cumulative ranking curve (SUCRA) values, as well as modified approaches used to present summary findings from meta-analyses.*	Materials and Method / Outcomes
Planned methods of analysis	14	Describe the methods of handling data and combining results of studies for each network meta-analysis. This should include, but not be limited to:<br>• *Handling of multi-arm trials;*<br>• *Selection of variance structure;*<br>• *Selection of prior distributions in Bayesian analyses; and*<br>• *Assessment of model fit.*	Materials and Method / Statistical analyses
**Assessment of Inconsistency**	**S2**	Describe the statistical methods used to evaluate the agreement of direct and indirect evidence in the treatment network(s) studied. Describe efforts taken to address its presence when found.	Materials and Method / Statistical analyses
Risk of bias across studies	15	Specify any assessment of risk of bias that may affect the cumulative evidence (e.g., publication bias, selective reporting within studies).	Materials and Method / Publication bias
Additional analyses	16	Describe methods of additional analyses if done, indicating which were pre-specified. This may include, but not be limited to, the following:<br>• Sensitivity or subgroup analyses;<br>• Meta-regression analyses;<br>• *Alternative formulations of the treatment network; and*<br>• *Use of alternative prior distributions for Bayesian analyses (if applicable).*_	Materials and Method / Sensitivity analyses
**RESULTS†**			
Study selection	17	Give numbers of studies screened, assessed for eligibility, and included in the review, with reasons for exclusions at each stage, ideally with a flow diagram.	Results / Study identification Figure 1, Table S2, Table S3
**Presentation of network structure**	**S3**	Provide a network graph of the included studies to enable visualization of the geometry of the treatment network.	Figure 2
**Summary of network geometry**	**S4**	Provide a brief overview of characteristics of the treatment network. This may include commentary on the abundance of trials and randomized patients for the different interventions and pairwise comparisons in the network, gaps of evidence in the treatment network, and potential biases reflected by the network structure.	Results / Network model formation / Figure 2

Study characteristics	18	For each study, present characteristics for which data were extracted (e.g., study size, PICOS, follow-up period) and provide the citations.	Table 1
Risk of bias within studies	19	Present data on risk of bias of each study and, if available, any outcome level assessment.	Table S4, Figure S1, Methodological quality
Results of individual studies	20	For all outcomes considered (benefits or harms), present, for each study: 1) simple summary data for each intervention group, and 2) effect estimates and confidence intervals. *Modified approaches may be needed to deal with information from larger networks.*	Table 1
Synthesis of results	21	Present results of each meta-analysis done, including confidence/credible intervals. *In larger networks, authors may focus on comparisons versus a particular comparator (e.g. placebo or standard care), with full findings presented in an appendix. League tables and forest plots may be considered to summarize pairwise comparisons.* If additional summary measures were explored (such as treatment rankings), these should also be presented.	Outcomes / Figure 3, Figure 4, Figure S2, Figure S3, Table 2
**Exploration for inconsistency**	**S5**	Describe results from investigations of inconsistency. This may include such information as measures of model fit to compare consistency and inconsistency models, *P* values from statistical tests, or summary of inconsistency estimates from different parts of the treatment network.	Inconsistency test Table S5, Table S6
Risk of bias across studies	22	Present results of any assessment of risk of bias across studies for the evidence base being studied.	Publication bias, Figure S6
Results of additional analyses	23	Give results of additional analyses, if done (e.g., sensitivity or subgroup analyses, meta-regression analyses, *alternative network geometries studied, alternative choice of prior distributions for Bayesian analyses,* and so forth).	Sensitivity analysis / Figure S4, Figure S5
**DISCUSSION**			
Summary of evidence	24	Summarize the main findings, including the strength of evidence for each main outcome; consider their relevance to key groups (e.g., healthcare providers, users, and policy-makers).	Discussion
Limitations	25	Discuss limitations at study and outcome level (e.g., risk of bias), and at review level (e.g., incomplete retrieval of identified research, reporting bias). *Comment on the validity of the assumptions, such as transitivity and consistency. Comment on any concerns regarding network geometry (e.g., avoidance of certain comparisons).*	Discussion
Conclusions	26	Provide a general interpretation of the results in the context of other evidence, and implications for future research.	Conclusion
**FUNDING**			
Funding	27	Describe sources of funding for the systematic review and other support (e.g., supply of data); role of funders for the systematic review. This should also include information regarding whether funding has been received from manufacturers of treatments in the network and/or whether some of the authors are content experts with professional conflicts of interest that could affect use of treatments in the network.	Funding

PICOS = population, intervention, comparators, outcomes, study design.

* Text in italics indicate wording specific to reporting of network meta-analyses that has been added to guidance from the PRISMA statement.

† Authors may wish to plan for use of appendices to present all relevant information in full detail for items in this section.

Table S2. Keywords and search results in different database.

Database	Keyword	Date	Results
PubMed	(Advanced cancer OR Metastasis OR Terminal illness) AND (End of life OR Palliative care) AND (quality of life OR QoL) AND (Physical Activity OR exercises OR physical activity OR yoga OR aerobic) AND (random OR randomized OR randomized)	2024/02/14	98
Embase	(Advanced cancer OR Metastasis OR Terminal illness) AND (End of life OR Palliative care) AND (quality of life OR QoL) AND (Physical Activity OR exercises OR physical activity OR yoga OR aerobic) AND (random OR randomized OR randomized)	2024/02/14	400
Web of Science	(Advanced cancer OR Metastasis OR Terminal illness) AND (End of life OR Palliative care) AND (quality of life OR QoL) AND (Physical Activity OR exercises OR physical activity OR yoga OR aerobic) AND (random OR randomized OR randomized)	2024/02/14	123
Cochrane review	(Advanced cancer OR Metastasis OR Terminal illness) AND (End of life OR Palliative care) AND (quality of life OR QoL) AND (Physical Activity OR exercises OR physical activity OR yoga OR aerobic) AND (random OR randomized OR randomized)	2024/02/14	10
Cochrane central	(Advanced cancer OR Metastasis OR Terminal illness) AND (End of life OR Palliative care) AND (quality of life OR QoL) AND (Physical Activity OR exercises OR physical activity OR yoga OR aerobic) AND (random OR randomized OR randomized)	2024/02/14	228

Table S3. Excluded article and reason.

Title	Excluded
Physical exercise and fatigue in advanced gastrointestinal cancer during chemotherapy	Incomplete data
Nurse-Supervised Exercise for People with Stage IV Cancer: The EFICANCER Randomized Clinical Trial	No baseline data
Effects of Walking Combined With Resistance Band Exercises on Alleviating Cancer-Related Fatigue	No quality-of-life data
Combination Therapy of Physical Activity and Dexamethasone for Cancer-Related Fatigue: A Phase II Randomized Double-Blind Controlled Trial	non exercise intervention
Resistance training in advanced cancer: a phase II safety and feasibility trial-home versus hospital	No quality-of-life data
Pilot Study of Effects of Yoga Therapy & Qigong for Pain, Sleep & Mood among ER+ Breast Cancer Survivors	No quality-of-life data
Impact of locally advanced head and neck cancer treatment: is there a role for exercise? FIT4TREAT	Incomplete data
Evaluating a web- and telephone-based personalised exercise intervention for individuals living with metastatic prostate cancer (ExerciseGuide): protocol for a pilot randomised controlled trial	Protocol only
Physical Activity for Symptom Management in Women With Metastatic Breast Cancer: A Randomized Feasibility Trial on Physical Activity and Breast Metastases	Functional QOL scale
A randomized phase II study of nutritional and exercise treatment for elderly patients with advanced non-small cell lung or pancreatic cancer: the NEXTAC-TWO study protocol	non exercise intervention
Pre-radiotherapy daily exercise training in non-small cell lung cancer: A feasibility study	Pretreatment intervention
ACE: advanced Cancer & Cachexia Exercise Trial	Protocol only
The HOPE Pilot Study: Harnessing Patient-Reported Outcomes and Biometric Data to Enhance Cancer Care	Not RCT
PR Effects of physical exercise in non-operable lung cancer patients undergoing palliative treatment	No quality-of-life data
Effects of nutrition and physical exercise intervention in palliative cancer patients: A randomized controlled trial	non exercise intervention
Exercise behavior and physical fitness in patients with advanced lung cancer	Not RCT
TIRED: a randomized controlled trial evaluating efficacy of cognitive behavior therapy and graded exercise therapy in severely fatigue patients with advanced cancer	Incomplete data
Effect of Exercise and Nutrition Prehabilitation on Functional Capacit in Esophagogastric cancer Surgery A Randomized Clinical Trial	non exercise intervention
Exercise Preserves Physical Function in Prostate Cancer Patients with Bone Metastases	No quality-of-life data
Walk this way: learning from a feasibility RCT of a walking intervention for people with recurrent and metastatic cancer	No quality-of-life data
Study protocol of the TIRED study: a randomized controlled trial comparing either graded exercise therapy for severe fatigue or cognitive behavior therapy with usual care in patients with incurable cancer	Protocol only
Exercise Intervention in Pediatric Patients with Solid Tumors: The Physical Activity in Pediatric Cancer Trial	Not advanced stage
Physical activity intervention for patients with advanced pancreatic cancer	Incomplete data
POSITIVE study: physical exercise program in non-operable lung cancer patients undergoing palliative treatment	Protocol only
Design of a randomized intervention study: the effect of dumbbell exercise therapy on physical activity and quality of life among breast cancer survivors in Malaysia	Protocol only
Physical exercise program in non-operable lung cancer patients undergoing palliative treatment-preliminary report of recruitment rates and feasibility of the POSITIVE study (part III)	Protocol only

Feasibility and Impact of a Physical Exercise Program in Patients with Advanced Cancer: A Pilot Study	Not RCT
Exercise in Patients with Non-Small Cell Lung Cancer	Not RCT
The effect of resistance exercise training on health-related quality of life of head-and-neck cancer patients undergoing definitive chemoradiation: results of a pilot study	Not RCT
"EXHALE": exercise as a strategy for rehabilitation in advanced stage lung cancer patients: a randomized clinical trial comparing the effects of 12 weeks supervised exercise intervention versus usual care for advanced stage lung cancer patients	Protocol only
The tired [treatment of fatigue during palliative care for advanced or metastatic disease] study: two interventions for fatigued advanced cancer patients, a randomized controlled trial	Protocol only
A randomized controlled trial on the effectiveness of strength training on clinical and muscle cellular outcomes in patients with prostate cancer during androgen deprivation therapy: rationale and design	No quality-of-life data
Effects of Physical Therapy on Pain and Mood in Patients with Terminal Cancer: A Pilot Randomized Clinical Trial	No quality-of-life data
Physical Exercise for Cancer Patients with Advanced Disease: A Randomized Controlled Trial	No quality-of-life data
Physical activity, quality of life, and the interest in physical exercise programs in patients undergoing palliative chemotherapy	Not RCT
Efficacy and safety of a modular multi-modal exercise program in prostate cancer patients with bone metastases: a randomized controlled trial	Protocol only
Combined Resistance and Aerobic Exercise Program Reverses Muscle Loss in Men Undergoing Androgen Suppression Therapy for Prostate Cancer Without Bone Metastases: A Randomized Controlled Trial	Not advanced stage
A phase III clinical trial of exercise modalities on treatment side-effects in men receiving therapy for prostate cancer	Protocol only
The effect of a physical exercise program in palliative care: A Phase II study	Not RCT

Table S4. Detailed quality assessment of included studies using Cochrane risk of bias 2 tool.

Author	Randomization process				Deviations from intended interventions								Missing outcome data					Measurement of the outcome						Selection of the reported result				Overall risk of bias
	1.1	1.2	1.3	1.0	2.1	2.2	2.3	2.4	2.5	2.6	2.7	2.0	3.1	3.2	3.3	3.4	3.0	4.1	4.2	4.3	4.4	4.5	4.0	5.1	5.2	5.3	5.0	6.0
Neuzillet et al. (2023) [26]	Y	Y	N	Low	Y	Y	PN	NA	NA	PY	NA	Low	PY	NA	NA	NA	Low	N	PN	Y	Y	PY	High	PY	PY	PN	High	High
Zimmer et al. (2018) [27]	Y	Y	N	Low	Y	Y	PN	NA	NA	Y	NA	Low	PN	PY	NA	NA	Low	PN	PN	Y	N	NA	Low	Y	PN	PN	Low	Low
Grote et al. (2018) [28]	Y	PY	PN	Low	Y	Y	PN	NA	NA	PY	NA	Low	Y	NA	NA	NA	Low	N	PN	Y	PY	PY	High	Y	PN	PN	Low	High
Tsianakas et al. (2016) [29]	Y	PY	PN	Low	Y	Y	PN	NA	NA	PY	NA	Low	N	Y	NA	NA	Low	N	PN	Y	Y	PN	Some concerns	PY	PN	PN	Low	Some concerns
Jensen et al. (2014) [30]	NI	NI	PN	Some concerns	Y	Y	PN	NA	NA	Y	NA	Low	N	PN	PN	NA	Low	PN	PN	Y	PN	NA	Low	Y	PN	PN	Low	Some concerns
Cheville et al. (2012) [31]	Y	Y	PN	Low	Y	Y	PN	NA	NA	Y	NA	Low	PN	PN	PN	NA	Low	PN	PN	Y	Y	PN	Some concerns	Y	PN	PN	Low	Some concerns
Hwang (2012) [32]	Y	Y	PN	Low	Y	PY	Y	PY	PY	NI	PY	High	PY	NA	NA	NA	Low	PN	PN	PN	NA	NA	Low	Y	PN	PN	Low	High
Ligibel et al. (2016) [33]	NI	NI	PN	Some concerns	Y	PY	PN	NA	NA	NI	PN	Some concerns	PY	NA	NA	NA	Low	PN	PN	Y	PY	PN	Some concerns	Y	PN	PN	Low	Some concerns
Henke et al. (2014) [34]	Y	Y	PN	Low	Y	Y	PN	NA	NA	NI	PN	Some concerns	N	PN	PN	NA	Low	PN	PN	Y	PN	NA	Low	PY	PN	PN	Low	Some concerns
Adamsen et al. (2009) [35]	Y	PY	PN	Low	Y	NI	NI	NA	NA	PY	NA	Some concerns	PY	NA	NA	NA	Low	PN	PN	N	NA	NA	Low	Y	PN	PN	Low	Some concerns
Cormie et al. (2013) [36]	PY	PY	PN	Low	Y	N	N	NA	NA	PN	N	Some concerns	Y	NA	NA	NA	Low	N	PN	N	NA	NA	Low	Y	PN	PN	Low	Some concerns

Table S5. Inconsistent test results of the standardized mean difference in quality of life improvement for advanced-stage cancer patients after exercise.

Comparison	Studies	NMA	Direct	Indirect	Difference	95CIL	95CIU	*p*-value
Aerobic : Aerobic + Strength	0	0.24	NA	0.24	NA	NA	NA	NA
Aerobic : Control	4	0.30	0.33	-0.06	0.40	-0.73	1.53	0.49
Aerobic : Strength	1	0.17	-0.05	0.34	-0.40	-1.53	0.73	0.49
Aerobic + Strength : Control	4	0.07	0.07	NA	NA	NA	NA	NA
Aerobic + Strength : Strength	0	-0.06	NA	-0.06	NA	NA	NA	NA
Strength : Control	2	0.13	-0.01	0.39	-0.40	-1.53	0.73	0.49

95CIL: lower limit of 95% confidence interval; 95CIU: upper limit of 95% confidence interval; NMA: network meta-analysis.

Table S6. Inconsistent test results for risk difference of dropout when applying a exercise program to improve the quality of life in advance stage cancer patients.

Comparison	Studies	NMA	Direct	Indirect	Difference	95CIL	95CIU	p-value
Aerobic : Strength	1	0.05	0.08	0.03	0.05	-0.34	0.43	0.81
Aerobic : Aerobic + Strength	0	0.01	NA	0.01	NA	NA	NA	NA
Aerobic : Control	4	0.01	0.01	0.06	-0.05	-0.43	0.34	0.81
Strength : Aerobic + Strength	0	-0.03	NA	-0.03	NA	NA	NA	NA
Strength : Control	2	-0.03	-0.02	-0.07	0.05	-0.34	0.43	0.81
Aerobic + Strength : Control	4	0.00	0.00	NA	NA	NA	NA	NA

95CIL: lower limit of 95% confidence interval; 95CIU: upper limit of 95% confidence interval; NMA: network meta-analysis

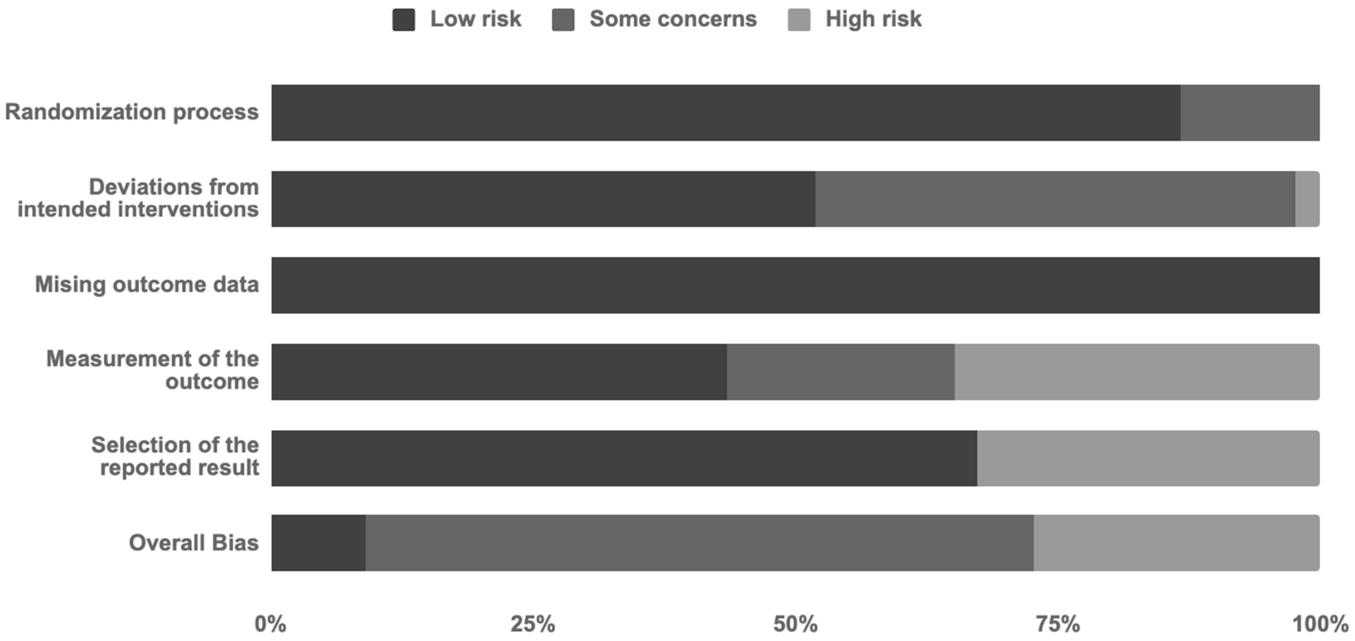

Figure S1. Summary of quality assessment for the studies included in the current network meta-analysis using version 2 of the Cochrane risk-of-bias tool for randomized controlled trials. Over half of the studies received some risks in the intervention adherence domain. This is because the interventions in these studies were markedly different, which could affect adherence and outcomes.

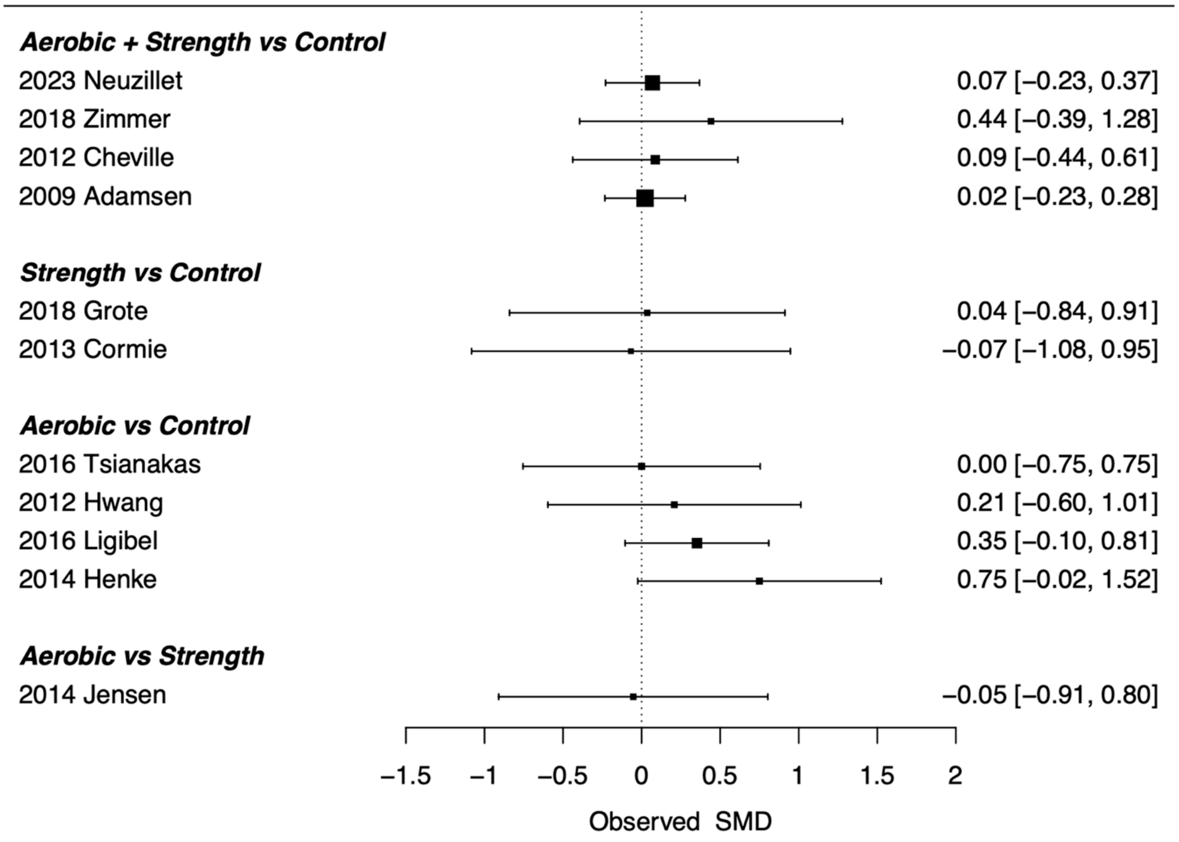

Figure S2. The forest plot of pair-wise comparisons for different exercise interventions in advanved stage cancer survivors to improve quality of life, retrieved from the included trials, demonstrates the standardized mean difference (SMD) of quality of life.

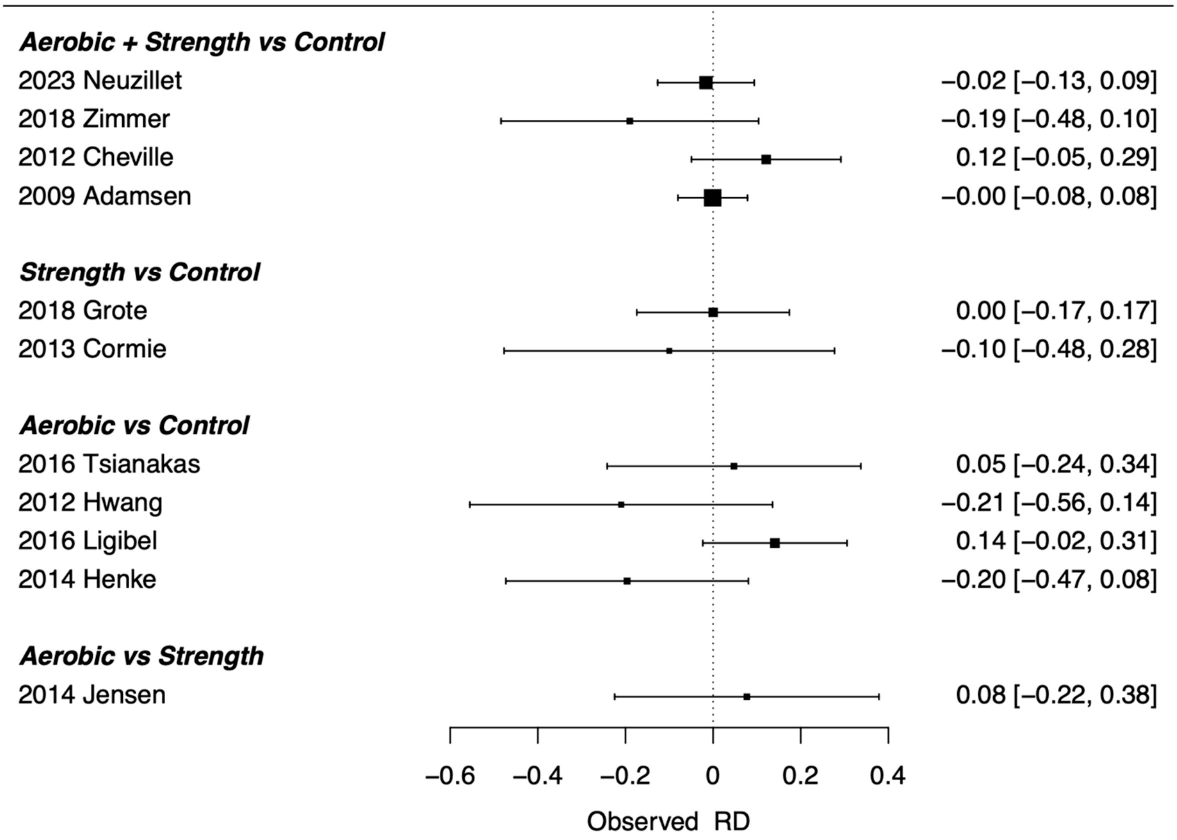

Figure S3. The forest plot of pair-wise comparisons for different exercise interventions in advanced stage cancer survivors to improve quality of life, retrieved from the included trials, demonstrates the risk difference (RD) of dropout rates. None of the comparisons reached statistical significance.

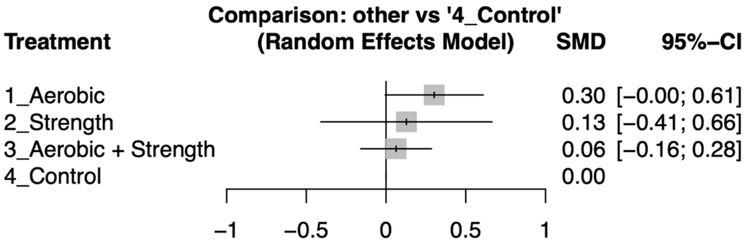

(**a**)

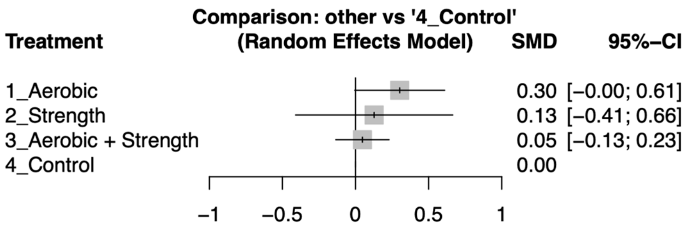

(**b**)

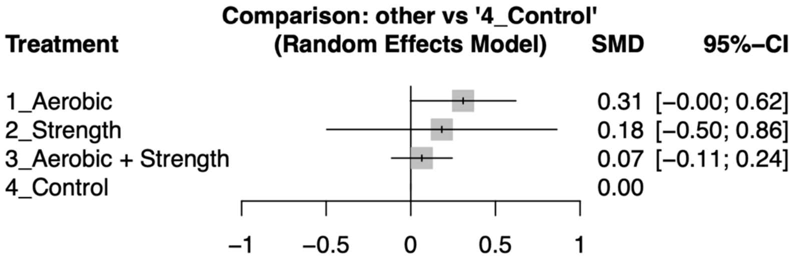
Comparison: other vs '4_Control'
Treatment
(Random Effects Model)
SMD
95%−CI
1_Aerobic 0.31 [−0.00; 0.62]
2_Strength 0.18 [−0.50; 0.86]
3_Aerobic + Strength 0.07 [−0.11; 0.24]
4_Control 0.00
−1 −0.5 0 0.5 1

(**c**)

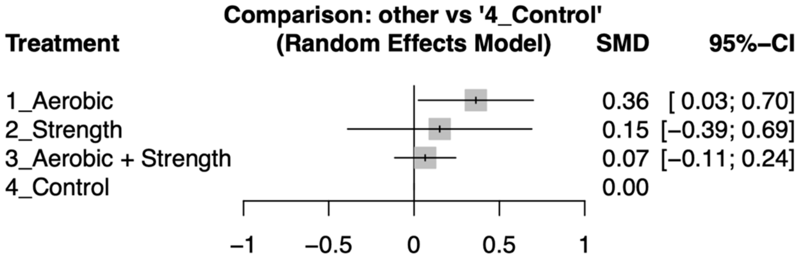
Comparison: other vs '4_Control'
Treatment
(Random Effects Model)
SMD
95%−CI
1_Aerobic 0.36 [0.03; 0.70]
2_Strength 0.15 [−0.39; 0.69]
3_Aerobic + Strength 0.07 [−0.11; 0.24]
4_Control 0.00
−1 −0.5 0 0.5 1

(**d**)

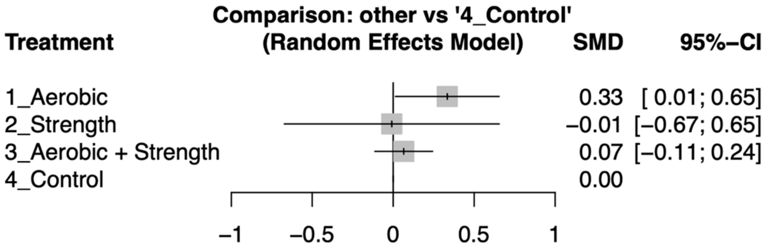
Comparison: other vs '4_Control'
Treatment
(Random Effects Model)
SMD
95%−CI
1_Aerobic 0.33 [0.01; 0.65]
2_Strength −0.01 [−0.67; 0.65]
3_Aerobic + Strength 0.07 [−0.11; 0.24]
4_Control 0.00
−1 −0.5 0 0.5 1

(**e**)

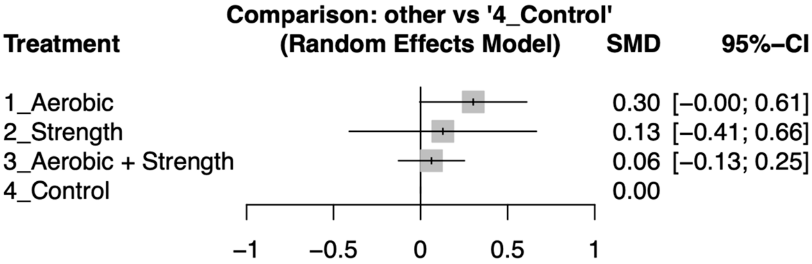
Comparison: other vs '4_Control'
Treatment
(Random Effects Model)
SMD
95%−CI
1_Aerobic 0.30 [−0.00; 0.61]
2_Strength 0.13 [−0.41; 0.66]
3_Aerobic + Strength 0.06 [−0.13; 0.25]
4_Control 0.00
−1 −0.5 0 0.5 1

(**f**)

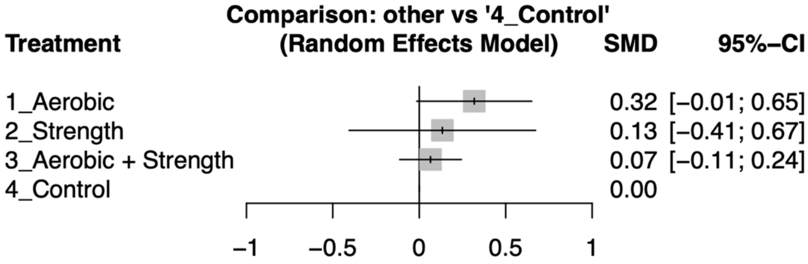
Comparison: other vs '4_Control'
Treatment
(Random Effects Model)
SMD
95%−CI
1_Aerobic 0.32 [−0.01; 0.65]
2_Strength 0.13 [−0.41; 0.67]
3_Aerobic + Strength 0.07 [−0.11; 0.24]
4_Control 0.00
−1 −0.5 0 0.5 1

(**g**)

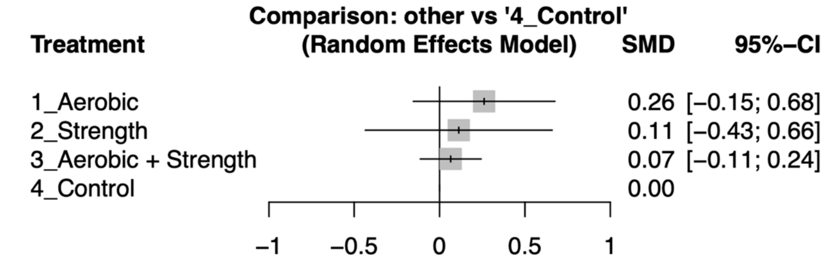

(**h**)

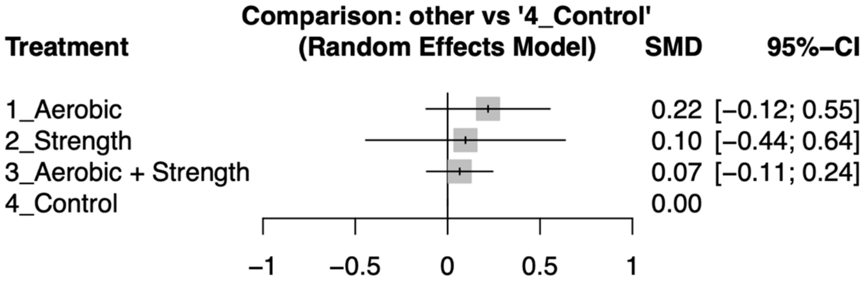

(**i**)

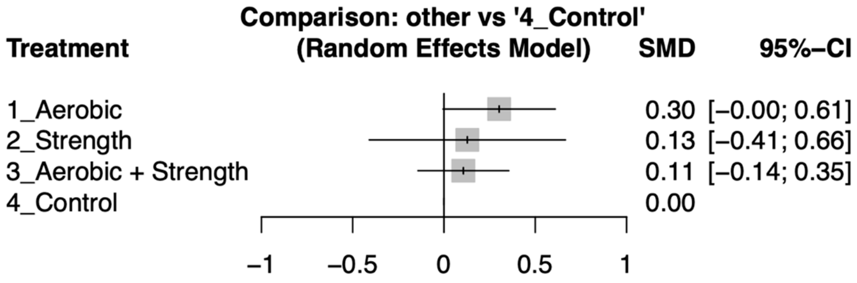

(**j**)

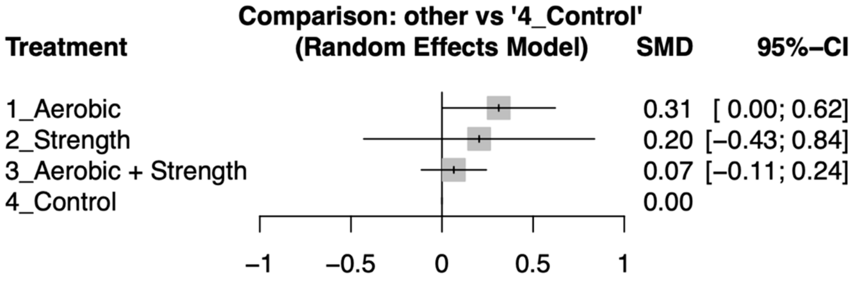

(**k**)

Figure S4. Forest plots are presented for sensitivity analysis with the one-study removal method, where each of the nine included studies was sequentially removed based on their year of publication (labeled from a to k). The rankings and clinical implications remained unchanged, indicating that the inclusion or exclusion of any single study does not alter the conclusions of our study.

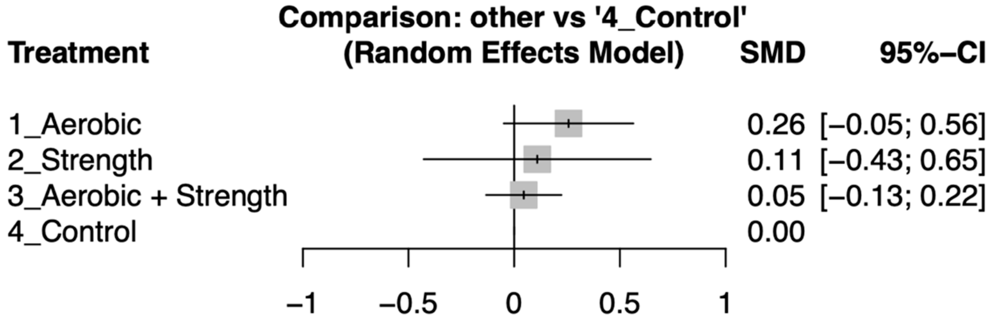

Figure S5. Forest plot displaying improved quality of life in advanced-stage cancer survivors after receiving different exercise interventions, presented as standardized mean differences (SMDs). The pre-post correlation coefficient used in the data calculation was changed from 0.8, used in Figure 3, to 0.5 in this figure as a sensitivity analysis. The ranking and clinical interpretations remained unchanged compared to Figure 3. This suggests that the conclusions of our study remain unchanged despite different assumptions regarding the coefficient used for transformation.

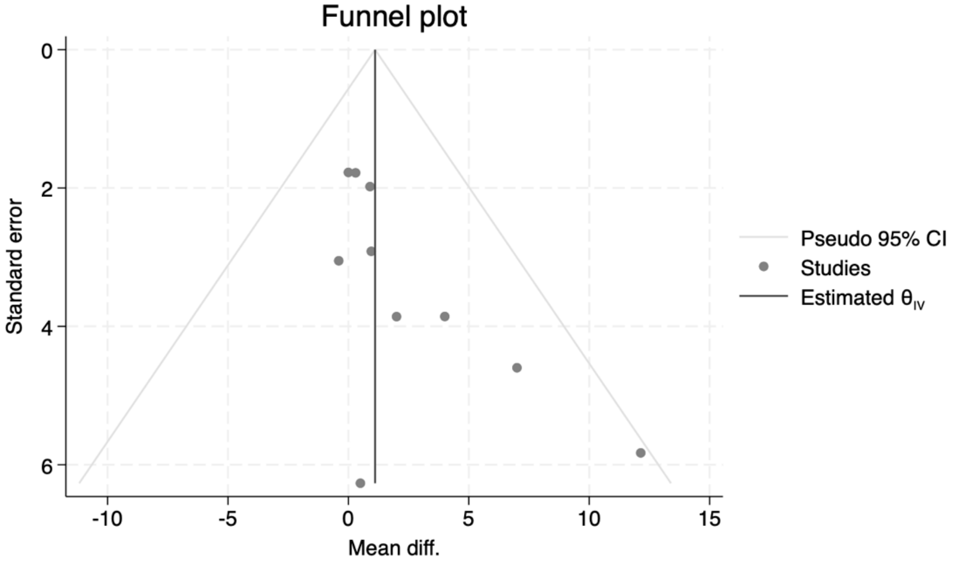

Figure S6. Funnel plot of all paired comparisons involving the common comparator, control group. The Egger's test yielded a *p*-value of 0.06, indicating no significant publication bias.
